# MyD88/CD40-based inducible co-stimulation to improve CAR T cell therapy

**DOI:** 10.1186/2051-1426-3-S2-P118

**Published:** 2015-11-04

**Authors:** Melinda Mata, Claudia Gerken, David M Spencer, Stephen Gottschalk

**Affiliations:** 1 Baylor College of Medicine, Houston, TX, USA; 2 Bellicum Pharmaceuticals and Baylor College of Medicine, Houston, TX, USA; 3 Department of Pediatrics, Center for Cell and Gene Therapy, Baylor College of Medicine, Houston, TX, USA

## Background

Adoptive immunotherapy with genetically modified T cells holds promise in improving outcomes for cancer patients. While a broad array of genetic modification strategies are being explored, few allow for the specific manipulation of adoptively transferred T cells *in vivo*. One successful example includes the introduction of an inducible ‘suicide gene’ to enable selective T-cell killing in the event of toxicities. Given the limited antitumor activity of adoptively transferred T cells for solid tumors in early clinical studies, we reasoned that introducing an inducible co-stimulatory molecule into T cells would allow for the selective activation of adoptively transferred T cells *in vivo* resulting in enhanced antitumor activity. Due to the role of MyD88 and CD40 signaling pathways to fine tune T-cell activation, and the recent success of using inducible (i) MyD88 and CD40 molecules to activate antigen-presenting cells, the goal of this project was to explore if T cells can be activated with iMyD88 and/or iCD40 molecules.

## Methods/results

We constructed a panel of retroviral vectors encoding mOrange as a marker gene and an inducible co-stimulatory molecule (iCO-STIM) consisting of a myristoylation tag, two FKBP dimerizer domains, and i) MyD88, ii) CD40, or iii) MyD88 + CD40. T cells expressing iMyD88, iCD40, or iMyD88.CD40 were generated by retroviral transduction, and transduction was confirmed by FACS analysis and Western blot. T cells expressing iCO-STIMs were activated with the CD3 monoclonal antibody OKT3 in the presence or absence of the chemical inducer of dimerization (CID), AP20187. iMyD88.CD40 T cells secreted the highest amount of IL2 in the presence of OKT3 + CID in comparison to iMyD88 or iCD40 T cells. To evaluate if activating iMyD88.CD40 in CAR T cells also enhances cytokine production, we generated T cells that expressed HER2-CARs and iMyD88.CD40 (HER2-CAR/iCO-STIM T cells). CID enhanced IL2 production of HER2-CAR/iCOSTIM T cells that were either stimulated with recombinant HER2 protein or HER2+ cell lines. Enhanced IL2 production was observed with 1st, 2nd, and 3rd generation HER2-CARs.

## Conclusion

We have generated CAR T cells with an inducible co-stimulatory molecule based on MyD88 and CD40. Preliminary functional analysis of CAR/iCO-STIM T cells is encouraging, warranting further active exploration of this approach to improve current T-cell therapy approaches for cancer.

